# Transcriptional Subtyping and CD8 Immunohistochemistry Identifies Patients With Stage II and III Colorectal Cancer With Poor Prognosis Who Benefit From Adjuvant Chemotherapy

**DOI:** 10.1200/PO.17.00241

**Published:** 2018-06-13

**Authors:** Wendy L. Allen, Philip D. Dunne, Simon McDade, Enya Scanlon, Maurice Loughrey, Helen G. Coleman, Christopher McCann, Kristy McLaughlin, Zsuzsanna Nemeth, Najeeb Ashraf Syed, Puthen Veettil Jithesh, Ken Arthur, Richard Wilson, Vicky M. Coyle, Darragh McArt, Graeme I. Murray, Leslie Samuel, Paolo Nuciforo, Jose Jimenez, Guillem Argiles, Rodrigo Dienstmann, Josef Tabernero, Lucia Picariello, Luca Messerini, Stefania Nobili, Enrico Mini, Kieran Sheahan, Elizabeth Ryan, Patrick G. Johnston, Sandra Van Schaeybroeck, Mark Lawler, Daniel B. Longley

**Affiliations:** **Wendy L. Allen**, **Philip D. Dunne**, **Simon McDade**, **Enya Scanlon**, **Maurice Loughrey**, **Helen G. Coleman**, **Christopher McCann**, **Kristy McLaughlin**, **Zsuzsanna Nemeth**, **Ken Arthur**, **Richard Wilson**, **Vicky M. Coyle**, **Darragh McArt**, **Patrick G. Johnston**, **Sandra Van Schaeybroeck**, **Mark Lawler**, and **Daniel B. Longley**, Queen’s University Belfast, Belfast; **Graeme I. Murray** and **Leslie Samuel**, National Health Service Grampian, Aberdeen, United Kingdom; **Najeeb Ashraf Syed** and **Puthen Veettil Jithesh**, Sidra Medical and Research Center, Qatar; **Paolo Nuciforo**, **Jose Jimenez**, **Guillem Argiles**, **Rodrigo Dienstmann**, and **Josef Tabernero**, University Hospital Vall d’Hebron, Barcelona, Spain; **Lucia Picariello**, **Luca Messerini**, **Stefania Nobili**, and **Enrico Mini**, University of Florence, Florence, Italy; and **Kieran Sheahan** and **Elizabeth Ryan**, University College Dublin, Dublin, Ireland.

## Abstract

**Purpose:**

Transcriptomic profiling of colorectal cancer (CRC) has led to the identification of four consensus molecular subtypes (CMS1 to 4) that have prognostic value in stage II and III disease. More recently, the Colorectal Cancer Intrinsic Subtypes (CRIS) classification system has helped to define the biology specific to the epithelial component of colorectal tumors; however, the clinical value of these classification systems in the prediction of response to standard-of-care adjuvant chemotherapy remains unknown.

**Patients and Methods:**

Using samples from four European sites, we assembled a novel cohort of patients with stage II and III CRC (n = 156 samples) and performed transcriptomic profiling and targeted sequencing and generated a tissue microarray to enable integrated multiomics analyses. We also accessed data from two published cohorts of patients with stage II and III CRC: GSE39582 and GSE14333 (n = 479 and n = 185 samples, respectively).

**Results:**

The epithelial-rich CMS2 subtype of CRC benefitted significantly from treatment with adjuvant chemotherapy in both stage II and III disease (*P* = .02 and *P* < .001, respectively), whereas the CMS3 subtype significantly benefitted in stage III only (*P* = .001). After CRIS substratification of CMS2, we observed that only the CRIS-C subtype significantly benefitted from treatment with adjuvant chemotherapy in stage II and III disease (*P* = .0081 and *P* < .001, respectively), whereas the CRIS-D subtype significantly benefitted in stage III only (*P* = .0034). We also observed that CRIS-C patients with low levels of CD8^+^ tumor-infiltrating lymphocytes were most at risk for relapse in both stage II and III disease (log-rank *P* = .0031; hazard ratio, 12.18 [95% CI, 1.51 to 98.58]).

**Conclusion:**

Patient stratification using a combination of transcriptional subtyping and CD8 immunohistochemistry analyses is capable of identifying patients with poor prognostic stage II and III disease who benefit from adjuvant standard-of-care chemotherapy. These findings are particularly relevant for patients with stage II disease, where the overall benefit of adjuvant chemotherapy is marginal.

## INTRODUCTION

Colorectal cancer (CRC) has the third highest worldwide incidence.^1^ Although 75% of patients present with operable disease—mainly stages II and III—approximately 40% experience disease recurrence.^2^ Compared with surgery alone, adjuvant chemotherapy improves survival in only approximately 3% of patients with stage II disease, rising to 15% to 20% for those with stage III disease. Overall, there is a clear need for treatment-stratifying biomarkers in patients with stage II and III CRC.

Significant advances have been made in the molecular stratification of CRC, leading to the identification of four consensus molecular subtypes (CMS1 to 4).^3^ CMS1 is enriched for microsatellite instability, is immune rich, and correlates with good prognosis, and CMS4 is stromal rich, with high levels of cancer-associated fibroblasts, and has a relatively poor prognosis. CMS3 is defined by the activation of multiple metabolic pathways, potentially as a result of its enrichment for *KRAS* mutations.^4^ The epithelial-rich CMS2 is the largest group, accounting for approximately 40% of all tumors. Although the CMS classification provides valuable prognostic information for early-stage CRC, its usefulness in selecting patients for adjuvant chemotherapy is not clear.^5-7^ In addition, there are wide variations in clinical outcome within each CMS subtype, particularly CMS2; therefore, there is a clear need for refinement of this classification system.

To define the biology that specifically drives neoplastic epithelial cells, the colorectal cancer intrinsic subtypes (CRIS) classification system was developed.^6^ Using this approach, five cancer epithelium-specific subtypes (CRIS-A) were identified that, by focusing on tumor epithelium, can potentially identify aspects of neoplastic biology that would be masked by contributions from the tumor microenvironment when using the CMS approach.^8,9^ In this study, we explored the use of CMS and CRIS classifications to predict the response to standard-of-care adjuvant treatment.

## PATIENTS AND METHODS

### Development of a Multiomics Patient Cohort

Using a combination of transcriptome profiling, next-generation sequencing, and tissue microarray generation, we developed a multiomics stage II and III CRC cohort. This taxonomy cohort was assembled from an initial cohort of 363 patients with stage II and III disease from four European centers. Of these, 188 samples with > 50% tumor content passed quality control and were subjected to RNA and DNA analysis (Appendix).

### Transcriptomics

High-quality transcriptomics data were obtained for 156 of the 188 samples (Almac Xcel array; Almac Diagnostics, Craigavon, United Kingdom). Data analysis was performed using the R Statistical Package (version 3.4.1; https://www.r-project.org/foundation/). All CEL files were loaded into R and processed using the makecdfenv, affy, and limma packages. Residual technical batch effects were corrected using the Combat method (sva package), and data were deposited in the National Center for Biotechnology Information Gene Expression Omnibus repository (GSE103479). The clinical-pathologic details of this cohort are provided in Table 1.

**Table 1.**
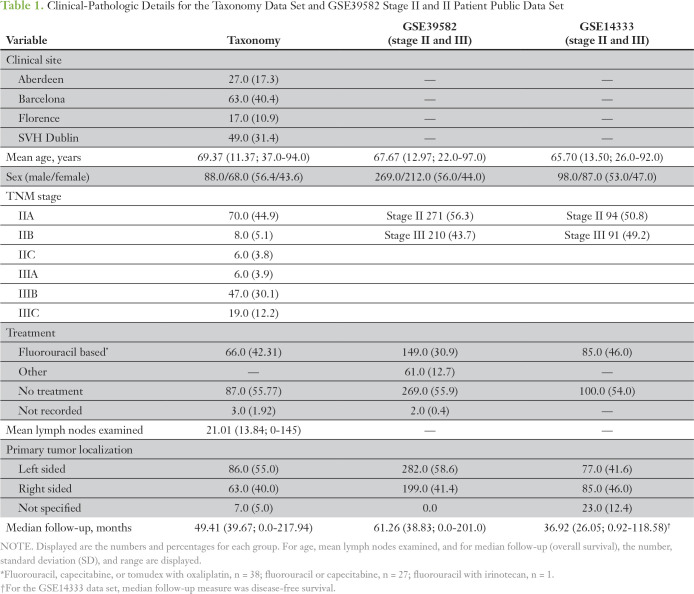
Clinical-Pathologic Details for the Taxonomy Data Set and GSE39582 Stage II and II Patient Public Data Set

### Data Analysis

GSE39582^10^ and GSE14333^11^ CRC data sets were downloaded from the National Center for Biotechnology Information Gene Expression Omnibus repository and their respective CEL files uploaded into R. The clinical-pathologic details of these cohorts are provided in Table 1. Each data set was subjected to CMS and CRIS classification. Kaplan-Meier estimators and Cox proportional hazards regression analysis were assessed using the survival and survminer R packages. Correlations between CMS and CRIS subtypes were assessed using Caleydo plots (Caleydo 3.1.5 software; www.caleydo.org).

### Tissue Microarray Construction

Tissue microarrays were generated with nine cores per tumor, incorporating three cores each from central tumor (CT), invasive front (IF) and tumor-adjacent stroma-rich (SR) regions. In addition, where available, three cores of adjacent normal colonic tissue were arrayed.

## RESULTS

### Molecular Subgroups

We initially assessed the proportion of CMS and CRIS subtypes^3,6,12,13^ (Data Supplement) that were present in our in-house multiomics taxonomy cohort (Table 1) and two other independent publicly available cohorts, GSE39582^10^ and GSE14333.^11^ These analyses revealed similar proportions of each CMS (Mann-Whitney paired *t* test, *P* value range .625 to 1.0) and CRIS subtype (Mann-Whitney paired *t* test, *P* value range .8125 to 1.0) compared with the published proportions of CMS and CRIS subtypes^3,6^ (Data Supplement).

### Benefit From Adjuvant Fluorouracil-Based Chemotherapy in CMS

**Fig 1.**
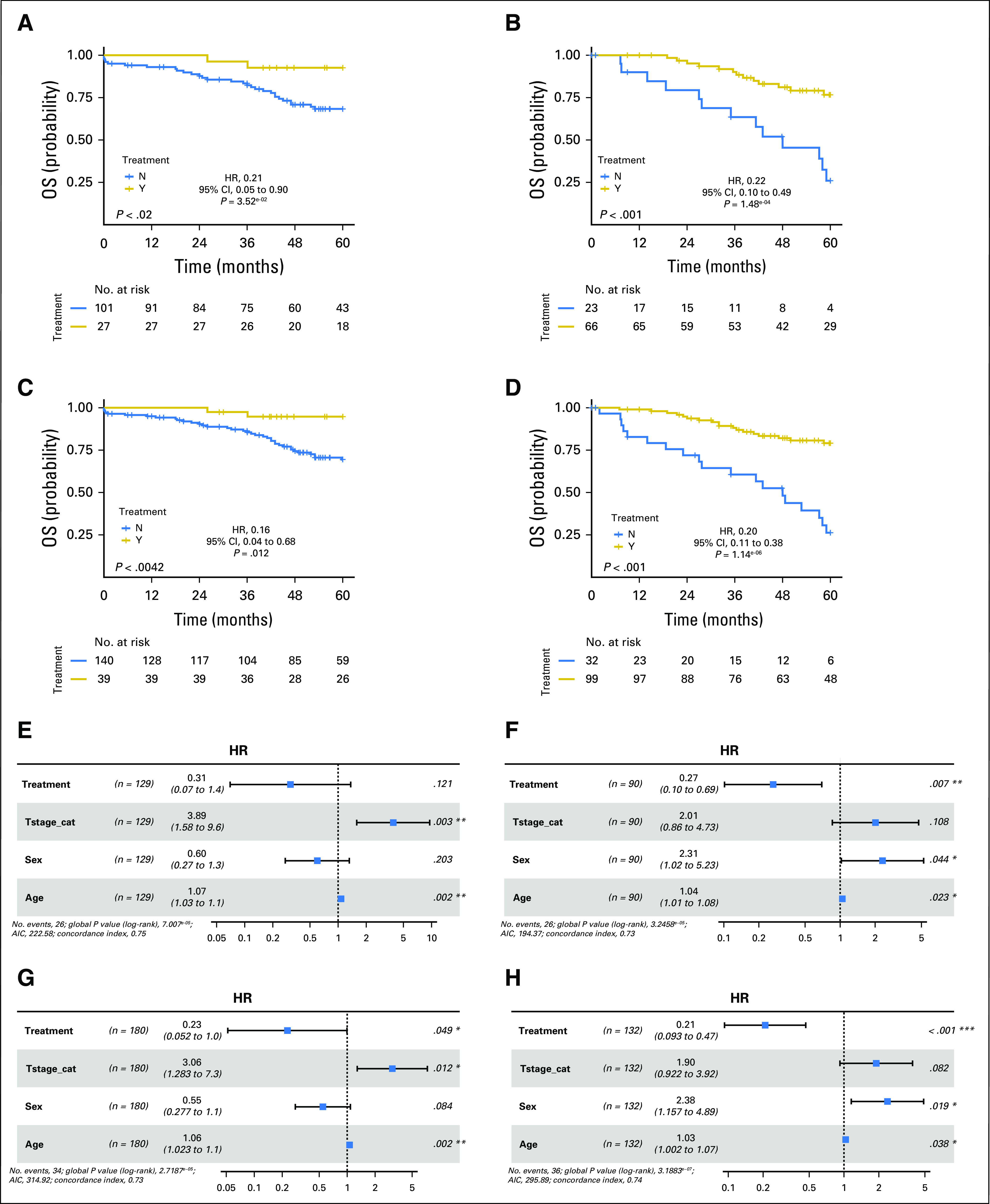
(A and B) Kaplan-Meier plots of 5-year overall survival (OS) for consensus molecular subtype 2 (CMS2) patients who received adjuvant fluorouracil (FU) -based treatment (gold) and those who did not receive treatment (surgery alone, blue), in the (A) stage II combined (taxonomy and GSE39582) cohort and the (B) stage III combined (taxonomy and GSE39582) cohort. (C and D) Kaplan-Meier plots for 5-year OS for combined CMS2 and CMS3 patients in the (C) stage II combined (taxonomy and GSE39582) cohort and the (D) stage III combined (taxonomy and GSE39582) cohort. Displayed is the log-rank test, along with the hazard ratio (HR) for the chemotherapy-treated group with 95% CIs and Wald test of significance. (E-H) Forest plots show the results from the adjusted Cox proportional hazards regression analysis for the (E) stage II CMS2 combined cohort, the (F) stage III CMS2 combined cohort, the (G) stage II CMS2 and CMS3 combined cohort, and the (H) stage III CMS2 and CMS3 combined cohort. Forest plots display the number of patients, HR for the chemotherapy-treated group with 95% CIs, and the Wald test of significance. The number of events and the log likelihood ratio is also displayed. For adjusted analyses, data are stratified by treatment and adjusted for T stage, sex, and age in stage II, and for age and sex in stage III. (*)Results defined as a significant hazard ratio in the Cox regression. AIC, Akaike's information criterion.

We used Kaplan-Meier analyses to determine the benefit from adjuvant fluorouracil (FU) -based chemotherapy in CMS1 to 4. In the taxonomy cohort, compared with patients who were treated with surgery alone, there were nonsignificant trends, particularly in stage III for CMS2 patients who received chemotherapy, for improved overall survival (log-rank test *P* = .13 and .056 for stage II and III, respectively; Data Supplement). Similar results were obtained in the larger GSE39582 cohort for stage II disease (log-rank test *P* = .071), whereas in stage III disease, this correlation reached significance (*P* = .001; Data Supplement). When we combined the taxonomy and GSE39582 cohorts to increase statistical power, the benefit from adjuvant chemotherapy for CMS2 was significant in both stage II disease (log-rank test *P* = .02; hazard ratio [HR], 0.21 [Wald test *P* = 3.52 × 10^−2^]; Fig 1A) and stage III disease (log-rank test *P* < .001; HR, 0.22 [Wald test *P* = 1.48 × 10^−4^]; Fig 1B). There was also significant benefit from adjuvant chemotherapy in the stage III CMS3 subtype (log-rank test *P* = .001; HR, 0.16 [Wald test *P* = 2.95 × 10^−3^]; Data Supplement) and a trend for benefit from chemotherapy in the stage II CMS3 subtype; however, this failed to reach significance (log-rank test *P* = .088; Data Supplement). Of note, no significant benefit from adjuvant chemotherapy was observed in CMS1 or CMS4, although a nonsignificant trend was observed in CMS4 stage III disease (log-rank test *P* = .089; Data Supplement).

These results suggest that the more epithelial CMS2 and CMS3 subgroups benefit from adjuvant chemotherapy. In support of this, in a combined analysis of CMS2 and CMS3, benefit from adjuvant chemotherapy was significant in both stage II disease (log-rank test *P* = .0042; HR, 0.16 [Wald test *P* = .012]; Fig 1C) and stage III disease (log-rank test *P* < .001; HR, 0.20 [Wald test *P* = 1.14 × 10^−6^]; Fig 1D). In contrast, in a combined analysis of the CMS1 and CMS4 subgroups, no benefit from adjuvant chemotherapy was observed (Data Supplement).

When CMS2 subgroup results for stage II disease were adjusted for T stage (T4 *v* T3), age, and sex using Cox proportional hazards regression analysis, the significance of the benefit from chemotherapy was lost (HR, 0.31; Wald test *P* = .121; log likelihood ratio, 7.0 × 10^−5^; Fig 1E); however, in stage III disease, adjusting for T stage (T4 or N2 *v* T1 to T3/N1), age, and sex, the significance of benefit from chemotherapy in CMS2 was maintained (HR, 0.27; Wald test *P* = .007; log likelihood *P* = 3.25 × 10^−5^; Fig 1F). In Cox proportional hazards regression analyses of the combined CMS2 and CMS3 subgroups, the significance of benefit from chemotherapy was maintained in stage II disease (HR, 0.23; Wald test *P* = .049; log likelihood ratio, 2.72 × 10^−5^; Fig 1G) and stage III disease (HR, 0.21; Wald test *P* < .001; log likelihood *P* = 3.19 × 10^−7^; Fig 1H).

### Clinical Implications of Tumor-Intrinsic Stratification in CMS2

**Fig 2.**
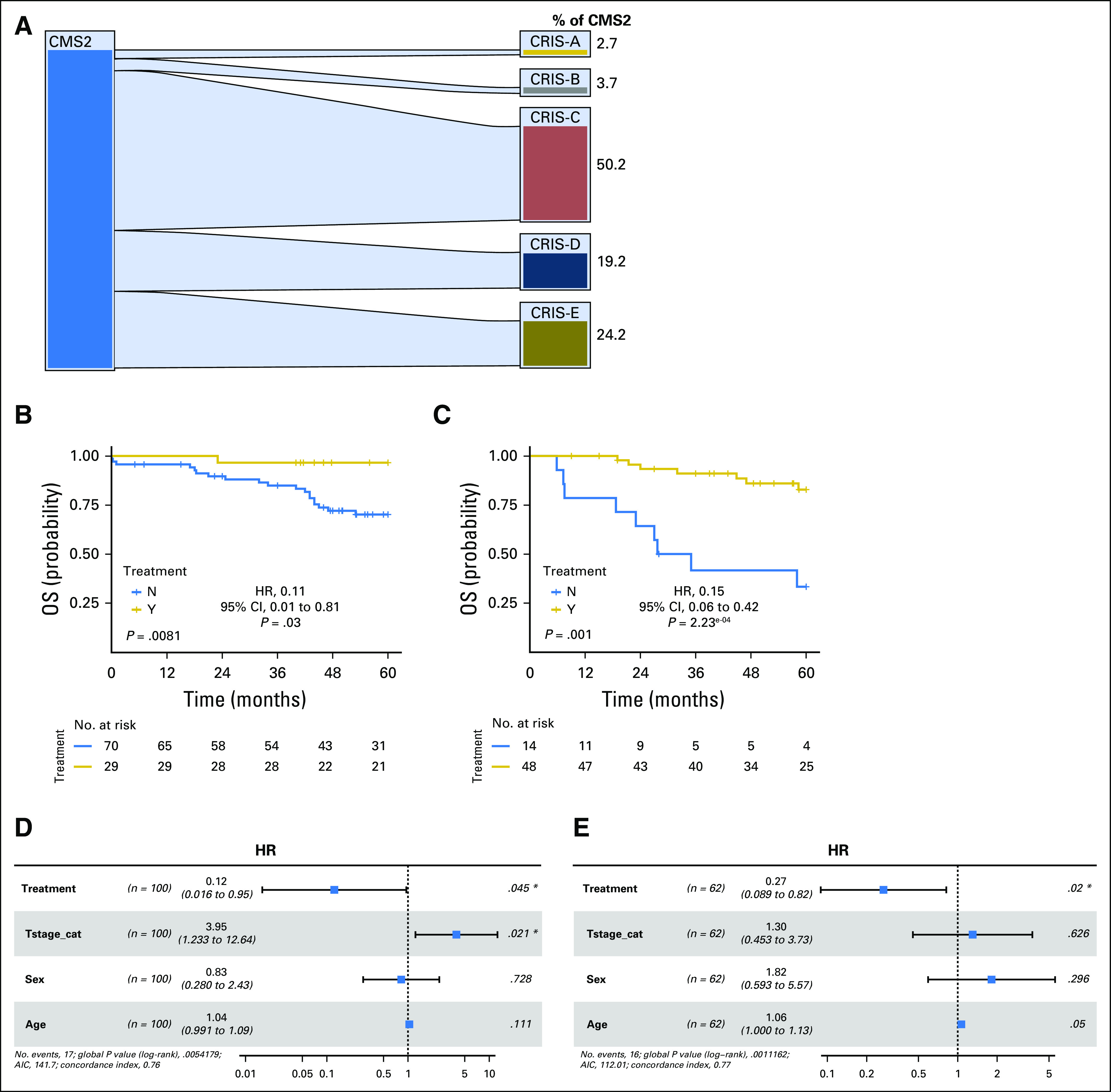
(A) Caleydo plots display mapping of patient samples from the consensus molecular subtype 2 to the Colorectal Cancer Intrinsic Subtypes (CRIS) in the combined (taxonomy and GSE39582) data sets. Plots were generated using Caleydo 3.1.5 software (www.caleydo.org). (B and C) Kaplan-Meier plots of 5-year overall survival (OS) for the (B) stage II CRIS-C combined cohort and the (C) stage III CRIS-C combined cohort. (D and E) Forest plots show results from the adjusted Cox proportional hazards regression analysis for (D) stage II and (E) stage III CRIS-C in the combined cohort. Forest plots display the number of patients, hazard ratio (HR) for the chemotherapy-treated group with 95% CIs, and the Wald test of significance. The number of events and the log likelihood ratio is also presented. (*)Results defined as a significant hazard ratio in the Cox regression. AIC, Akaike's information criterion.

As previously reported,^6^ there are limited associations between CMS and CRIS classifications—for example, CMS4 is distributed relatively evenly between the five CRIS subtypes (Data Supplement); however, some clear patterns were observed, with CMS2 almost exclusively distributed between CRIS-C, -D, and -E (Fig 2A). Subsequently, we investigated whether substratification of CMS2 into CRIS-C, -D, or -E could identify a more specific subset of patients with stage II and III disease who derive benefit from adjuvant chemotherapy. In the combined taxonomy/GSE39582 cohort, only the CRIS-C subgroup (the largest subgroup of CMS2: 50.2% (n = 110); Fig 2A) displayed significant benefit from chemotherapy in both stage II (log-rank test *P* = .0081; HR, 0.12 [Wald test *P* = .03]; Fig 2B) and stage III disease (log-rank test *P* < .001; HR, 0.15 [Wald test *P* = 2.23 × 10^−4^]; Fig 2C) or combined stage II and III (log-rank test *P* = 3.5 × 10^−4^; HR, 0.26 [Wald test *P* = 8.8 × 10^−4^]; Data Supplement). These results were confirmed in an additional independent cohort, GSE14333 (log-rank test *P* = .02; HR, 0.12 [Wald test *P* = .05]; Data Supplement). In contrast, there was no significant benefit from adjuvant chemotherapy in stage II CRIS-D patients (log-rank test *P* = .28) or in stage II and III CRIS-E patients (log-rank test *P* = .37 and *P* = .1, respectively); however, these analyses did reveal significant benefit from chemotherapy in stage III CRIS-D patients (log-rank test *P* = .0034; HR, 0.21 [Wald test *P* = 7.74 × 10^−3^]; Data Supplement). In the other 2 CRIS subgroups (CRIS-A and -B), no significant benefit from adjuvant chemotherapy was observed in either stage II or III disease, although a nonsignificant trend was observed in CRIS-A for stage III disease (*P* = .057; Data Supplement), which is consistent with this subgroup being enriched for CMS3, where a significant benefit was observed (Data Supplement). When CRIS-C results were adjusted for T stage, age, and sex using Cox proportional hazards regression analyses, the benefit from chemotherapy maintained significance in stage II disease (HR, 0.12; Wald test *P* = .045; log likelihood *P* = .0054; Fig 2D) and stage III disease (HR, 0.27; Wald test *P* = .02; log likelihood *P* = 1.11 × 10^−3^; Fig 2E); furthermore, for combined stage II and III disease, adjusted HR was 0.20 (Wald test *P* < .001; log likelihood *P* = 7.5 × 10^−6^; Data Supplement). These results indicate that CRIS-C classification predicts benefit from adjuvant chemotherapy in both patients with stage II and III disease independently of other clinicopathologic factors.

### Immunohistochemical Assessment of CD8^+^ Tumor-Infiltrating Lymphocytes

Although benefit from adjuvant chemotherapy for CRIS-C patients is clear in stage II and III disease (Figs 2A-2D), approximately 70% of stage II CRIS-C patients survive with surgery alone (Fig 2B). Thus, we sought to define a routine method that could identify high-risk stage II CRIS-C patients who should be administered adjuvant FU-based chemotherapy. Initially, we examined sequencing data from the taxonomy and GSE39582 cohorts. As previously reported,^6^ the CRIS-C subgroup is predominantly *TP53* mutant and *KRAS* wild type and almost exclusively *BRAF* wild type (Data Supplement). In line with this genotype, we also found that the CRIS-C subgroup is associated with left-sided tumors (Fisher's exact test: CRIS-C against all others, *P* = 6.27 × 10^−10^; 95% CI, 2.26 to 5.48; odds ratio, 3.48); however, stratifying CRIS-C patients on the basis of either *TP53* or *KRAS* mutational status did not identify CRIS-C patients who were at higher risk of relapse after surgery (data not shown).

**Fig 3.**
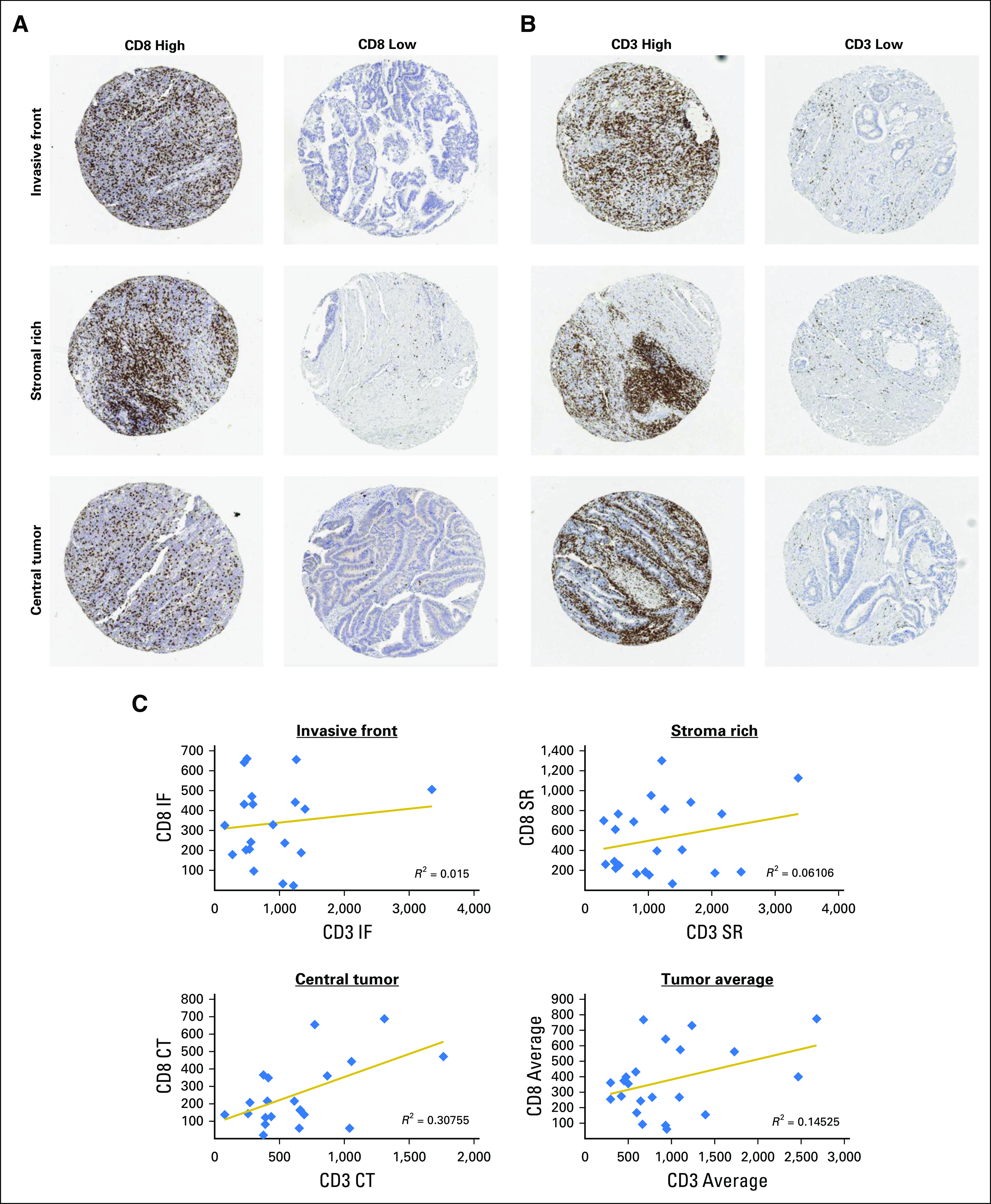
(A and B) Representative images of samples display high and low (A) CD8 and (B) CD3 expression in each of the three tumor regions sampled: invasive front (IF), stromal rich (SR), and central tumor (CT). (C) Correlations between CD8 and CD3 at each of the three regions sampled: IF, SR, and CT, as well as the average expression. Displayed are the equations of the line and *R*^2^ value for the correlation.

Given that patients with stage II and III CRC with high levels of T-cell infiltration have better prognoses,^2^ we next assessed whether T-cell infiltration could be used to distinguish between low- and high-risk CRIS-C patients. To account for potential intratumoral heterogeneity of putative biomarkers in specific tumor regions, TMAs were generated from the taxonomy cohort to incorporate three cores each from CT, IF, and tumor-adjacent SR regions. This unique design enables us to assess locoregional variations in biomarker expression. CD8 and CD3 levels were defined using immunohistochemistry, and patients were stratified into high and low groups using the median as cutoff (representative CD8 and CD3 images; Figs 3A and 3B). Of interest, correlations between CD3 and CD8 scores were relatively low (Fig 3C). In combined analyses of stage II and III disease, CRIS-C patients whose tumors had high levels of CD8^+^ lymphocytes in the IF, SR, and CT regions had significantly better overall survival than did those with low levels (log-rank test *P* = .023, *P* = .032, and *P* = .011, respectively; Data Supplement). We further assessed CD8^+^ lymphocytes in histologically normal tissue adjacent to the tumor and found no difference in survival between high and low CD8 levels (log-rank test *P* = .72; Data Supplement). Of note, no correlations were found between CD3^+^ lymphocyte levels and prognosis (IF log-rank test *P* = .55; SR log-rank test *P* = .75; CT log-rank test *P* = .8; normal log-rank test *P* = .82; Data Supplement).

**Fig 4.**
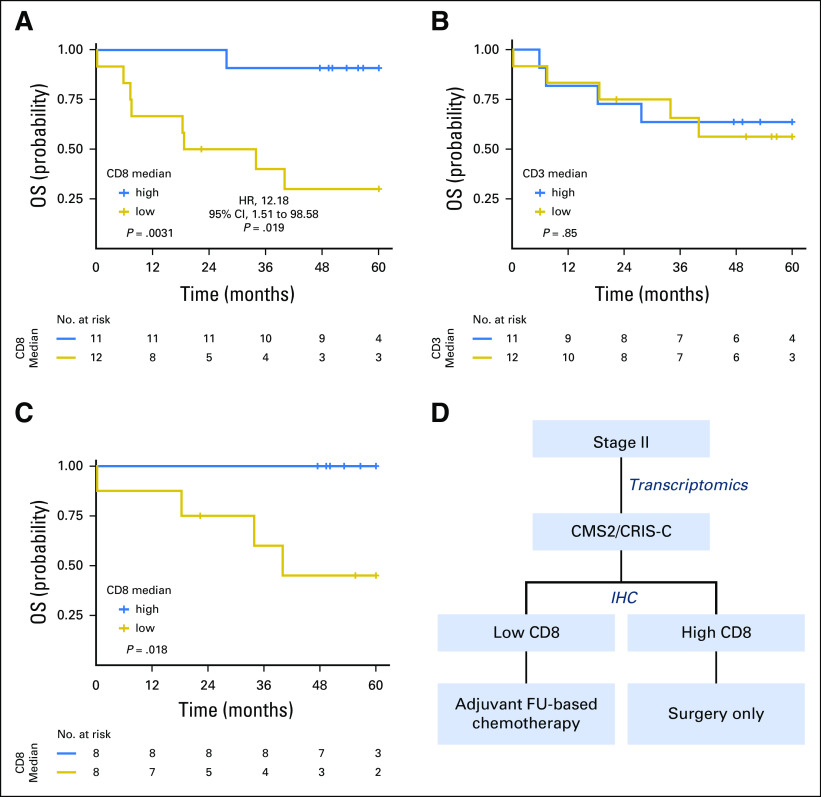
(A-C) Kaplan-Meier plots of patients with (A) CD8-high versus CD8-low stage II and III disease in the Colorectal Cancer Intrinsic Subtype (CRIS) -C surgery-only, taxonomy cohort; (B) CD3-high versus CD3-low stage II and III disease in the CRIS-C surgery-only, taxonomy cohort; and (C) CD8-high versus CD8-low stage II disease in the CRIS-C surgery-only, taxonomy cohort. Patients were split into high (blue) and low (gold) CD8 or CD3 groups using the median as cutoff. Significance was assessed using a log-rank test. (D) Diagram of a potential decision tree for treatment of patients with stage II disease in the CRIS-C cohort. CMS2, consensus molecular subtype 2; FU, fluorouracil; IHC, immunohistochemistry.

For each patient, CD8 scores in each tumor subregion correlated closely with one another (*P* < .001; Data Supplement). We therefore combined CD8 scores from each region where at least two of three cores were present per region—CT, IF, and SR—into an average score per patient. As expected on the basis of correlations for individual regions (Data Supplement), applying this tumor average score to the combined stage II and III taxonomy cohort revealed that high levels of CD8^+^ lymphocytes identified CRIS-C patients with good prognosis (log-rank *P* = .0031; HR, 12.18 [Wald test *P* = .0191]; Fig 4A). In contrast, average CD3 scores, which again did not correlate closely with average CD8 scores (Fig 3C), were not prognostic (Fig 4B). Of importance, the tumor average CD8 score also robustly risk stratified stage II CRIS-C patients (log-rank *P* = .018; Fig 4C). Of note, CD8 mRNA expression was unable to distinguish good and poor prognosis patients (log-rank *P* = .83; Data Supplement), which indicates the need for immunohistochemistry in combination with transcriptomic profiling for effective prognostication. Thus, CRIS-C patients with high levels of CD8^+^ lymphocytes have an excellent prognosis, which suggests that these patients do not require adjuvant chemotherapy. In contrast, as less than one half of patients with stage II CRIS-C/CD8 low tumors survive with surgery alone (Fig 4C), and given the significant benefit of FU-based chemotherapy in the CRIS-C subgroup (Fig 2), these data suggest that the CRIS-C/CD8 low subgroup should be treated with chemotherapy (Fig 4D).

## DISCUSSION

More than a decade ago, the seminal paper from Galon et al^14^ demonstrated the importance of antitumor immunity in CRC by showing the prognostic value of assessing immune infiltration. More recently, on the basis of gene expression patterns, it was proposed that CRC is composed of four distinct subtypes, called CMS.^3^ This classification incorporates gene expression profiles from tumor, stroma, and immune cells. Recently, an alternative transcriptomics-based classification was proposed that focuses on gene expression exclusively within the tumor cell compartment. This CRIS classification approach maps closely to the tumor’s underlying mutations.^6,15^

In this study, we highlight the potential clinical utility of CMS for selecting patients with stage III CRC for adjuvant chemotherapy, with a significant benefit from postsurgery chemotherapy observed in the epithelial-rich CMS2 and CMS3 subgroups—both alone and combined—but not in the more undifferentiated CMS1 or CMS4 subgroups.^16,17^ We show that additional stratification of CMS2 tumors into CRIS-C, CRIS-D, and CRIS-E identifies CRIS-C and CRIS-D as the subgroups of patients within CMS2 that derive a clear benefit from adjuvant chemotherapy in stage III disease.

In stage II CRC, use of chemotherapy after surgery is still a matter of debate. Therapeutic benefit from adjuvant chemotherapy is modest for this group as a whole, with an absolute improvement in survival of approximately 3%.^18-22^ Currently, additional pathologic characteristics, such as obstruction, perforation, extramural venous invasion, and T stage (T4), are used to identify poor prognostic stage II disease and guide the decision of whether to start chemotherapy treatment.^23^ Additional methods of assessing the risk of recurrence in the adjuvant disease setting have been the focus of many studies in recent years,^24-29^ which has led to the development of the 12-gene Oncotype DX assay.^30^ This algorithm has been extensively clinically tested^31-33^ and demonstrated to identify patients with stage II disease who are at higher risk of recurrence and patients with stage III disease who are at lower risk of recurrence.^34,35^ Additional signatures have been proposed, including the 18-gene prognostic classifier, known as ColoPrint,^36^ and the 634-gene prognostic classifier, known as ColDX.^27^ The current study indicates that the transcriptionally definable CMS2/CRIS-C patient subgroup may be the cohort of patients within stage II disease that benefits from standard adjuvant FU-based chemotherapy. Of importance, this benefit was found to be independent of T stage. Moreover, none of the other CRIS subgroups derived significant benefit from adjuvant chemotherapy in the stage II setting.

Subsequently, we found that low levels of tumoral/peritumoral CD8^+^ lymphoid cells could identify CRIS-C patients with stage II disease (and indeed stage III) who are most at risk for relapse after surgery and who therefore should be administered adjuvant chemotherapy. These data correlate well with our previous study on the prognostic significance of immune-derived programmed death ligand 1 mRNA expression in CRC, in which we postulated that patients with low immune infiltrates would significantly benefit from adjuvant FU-based chemotherapy after surgery.^9,37^ Meta-analysis of the IDEA (International Duration Evaluation of Adjuvant Chemotherapy) collaboration examined whether a 3-month duration of oxaliplatin-containing adjuvant chemotherapy—FOLFOX4, modified FOLFOX6, or XELOX—is as effective as a 6-month schedule in patients with stage III CRC. This study found that the 3-month treatment was almost as effective as the 6-month treatment and reduced the risk of treatment-associated toxicity, thus concluding that a 3-month treatment would be more beneficial for patients with low-risk (T1 to 3/N1 tumors) stage III disease.^38^ Our study suggests that levels of CD8^+^ lymphocytes could also be used to identify such low-risk patients, at least in the CRIS-C subgroup. Of note, CRIS-C is enriched for mutant *TP53* and wild-type *KRAS* tumors,^6^ but neither of these established molecular markers provided additional information with regard to disease outcome within the CRIS-C subgroup.

Collectively, these results provide the first evidence of the predictive value of the now well-established CMS and more recently described CRIS transcription-based classification systems. Our results also emphasize the utility of combining CMS and CRIS subtyping in a substratification strategy to maximize clinical benefit from adjuvant FU-based chemotherapy in patients with stage II and III CRC. In addition, CRIS classification, in combination with assessment of CD8 tumor-infiltrating lymphocytes, would potentially enable the prospective identification of the CRIS-C/CD8-low stage II patients who significantly benefit from adjuvant FU-based chemotherapy. However, we recognize that there are a number of limitations in the current study, which was conducted on a relatively small number of retrospective samples that were collected outside of clinical trials, and we realize that this hypothesis-generating study now requires validation in either larger patient cohorts or stratified trial cohorts enriched for the CRIS-C patient subtype. Nonetheless, this study suggests that transcription-based classification systems, such as CMS and CRIS, have the potential to be developed into patient stratification tools and, when used alone or alongside other molecular pathology approaches, such as immunohistochemistry, could enable the selection of patients with CRC who are most likely to benefit from adjuvant chemotherapy, while at the same time sparing nonresponders the potentially harmful treatment-related adverse events and sequelae of chemotherapy. Of importance, the CRIS subtyping method uses gene expression from tumor epithelial cells only and is independent of stromal-derived signals; therefore, the CRIS subgroups can be detected irrespective of the profiling technology used or the tissue source.^15^ Such robustness and reproducibility are critical for clinical translation. In conclusion, this study suggests that patients with stage III CRIS-C and stage II CRIS-C/CD8-low disease would benefit from adjuvant FU-based chemotherapy. This now requires additional validation in larger patient cohorts.

## Appendix

### Development of a Multiomics Patient Cohort

The taxonomy cohort was assembled from an initial cohort of 363 patients with stage II and III disease from four European Centers (Vall d’Hebron Institute of Oncology, Barcelona, Spain; St Vincent’s University Hospital, Dublin, Ireland; University of Florence, Florence, Italy; and University of Aberdeen, Aberdeen, United Kingdom). This work was approved by the Medicine, Dentistry, and Biomedical Sciences School Ethics Committee (ref: 12/12v4).

Samples from 194 patients with 50% or more tumor content as assessed by hematoxylin and eosin staining were subjected to RNA extraction. Of these, 188 samples passed quality control (spectrophotometer A260/280: 1.68 to 2.08; two distinct peaks 18S and 28S on a bioanalyzer).

### Data Analysis

GSE39582 and GSE14333 data sets represent 585 fresh frozen and 290 fresh frozen surgically resected primary tumor samples, respectively. Both data sets contain all stages (I to IV) and treated and untreated samples with accompanying clinical follow-up. Within the GSE39582 and GSE14333 data sets, only patients with stage II and III disease were selected for additional analysis—479 samples from GSE39582 and 185 samples from GSE14333.

The CMSclassifier R package was downloaded from github (https://github.com/Sage-Bionetworks/crcsc), and the CRISclassifier R package was downloaded from Isella et al^6^ and implemented using the Nearest Template Prediction method.

### Sequencing

Suitable-quality DNA was obtained for tumor samples (n = 188; ×500 mean coverage) and matched normal tissues (n = 128; ×100 mean coverage) that were then sequenced using a Roche/Nimblegen Seq-Cap-EZ panel (Roche, Mannheim, Germany) of 130 clinically relevant colorectal cancer genes. Sequencing data were made available as multiple libraries for each sample. Data for samples were preprocessed and aligned according to best practices (Broad Institute: https://gatkforums.broadinstitute.org/gatk/discussion/3060/how-should-i-pre-process-data-from-multiplexed-sequencing-and-multi-library-designs) using Burrows-Wheeler Aligner for alignment and GATK 3.4 for realignment and recalibration. For tumor-only samples, a panel of normals was generated using available normal samples. Mutect 3.1 (Cibulskis K, et al: Nat Biotechnol 31:213-219, 2013) was used for variant calling, and a variant effect predictor was used for annotation of filtered VCF files.

### CD3 and CD8 Immunohistochemistry and Scoring

CD3 was detected using the anti-CD3 antibody 2GV6 (Ventana Medical Systems, Tucson, AZ) on the BenchMark XT staining platform (Ventana). CD8 was detected using the anti-CD8 antibody C8/144B (Dako, Carpinteria, CA) using the Leica Bond Max staining platform (Leica Microsystems, Wetzlar, Germany). For CD8 evaluation, 74% of samples had triplicate cores for all three regions analyzed. An additional 11% of samples had two of their three cores available for analysis. For CD3 analysis, 88.9% of samples had triplicate cores for all three regions analyzed. An additional 3.4% of samples had two of their three cores present for analysis. For each tumor core, CD3^+^ and CD8^+^ T-cell populations were scored using the open access image analysis software QuPath (https://qupath.github.io; Bankhead P, et al: Sci Rep 7:16878, 2017). Measurements of tissue area and positive cell counts for each core provided cell density measurements, which are expressed as the number of positive cells per square millimeter of tissue. Subsequent analysis took a mean of each triplicate core where applicable. High and low CD3/CD8 levels were calculated using the median level as cutoff.

### Statistical Analyses

For Kaplan-Meier survival analysis and Cox proportional hazards regression analysis, significance was assessed using log-rank and Wald tests, respectively. In addition, we calculated the log likelihood ratio. In all cases, the survival end point measured was 5-year overall survival, unless otherwise stated. Cox proportional hazards regression analysis was used to assess overall survival at 5 years for adjuvant chemotherapy before and after adjustment for age, T stage, and sex (stage II and III) and age, sex, and stage (stage II and III combined). For correlation analysis, Pearson’s correlation coefficient was calculated with a two-tailed test of significance. Statistical analyses were calculated using the paired Mann-Whitney U test and Fisher's exact test, both two tailed. All statistical analyses were carried out in R, and *P* values < .05 indicated statistical significance.
